# Investigation of fluctuations in blood glucose level due to dietary restrictions during impacted mandibular third molar extraction under intravenous sedation: effect of perioperative glucose administration

**DOI:** 10.1007/s10006-020-00843-w

**Published:** 2020-04-27

**Authors:** Mio Sekine, Yuya Tomita, Asami Iguchi, Kazuyuki Fujii

**Affiliations:** 1 Anesthesiology and Resuscitation, The Nippon Dental University Graduate School of Life Dentistry at Niigata, Niigata, Japan; 2 Department of Dental Anesthesiology, The Nippon Dental University School of Life Dentistry at Niigata, 1-8 Hamauracho Chuoku, Niigata, 951-8580 Japan

**Keywords:** Glucose levels, Intravenous sedation, Local anesthesia, Impacted mandibular third molar, 5% glucose solution

## Abstract

**Objective:**

We aimed to investigate the usefulness of glucose administration for maintaining perioperative glycemic control in patients with dietary restrictions during 4 h prior to impacted mandibular third molar extraction under intravenous sedation.

**Methods:**

Fifty-four individuals scheduled to undergo extraction of impacted mandibular third molars under intravenous sedation, with preoperative blood glucose levels (GL) of 70–110 mg/dL, were evaluated and divided into 3 groups (*n* = 18 each): control group receiving glucose-free sodium lactate Ringer’s solution, perioperative GL group receiving 100 mL of 5% glucose solution immediately after local anesthesia, and postoperative GL group receiving 100 mL of 5% glucose solution immediately after surgery completion. Blood glucose levels, systolic blood pressure, diastolic blood pressure, and heart rate were measured.

**Results:**

Glucose levels of those in the control and perioperative GL groups decreased within the standard range 90 min after surgery, compared with the preoperative blood glucose level. However, in the postoperative GL group, glucose levels were similar to the preoperative levels. Systolic and diastolic blood pressure and heart rate were not affected by glucose administration, and sedation could be maintained without an invasive procedure.

**Conclusions:**

Following a restriction on eating and drinking 4 h prior to surgery, the blood glucose level gradually decreased in the perioperative period but remained within the reference range until 90 min following surgery. The administration of 100 mL 5% glucose solution immediately after surgery was sufficient for the prevention of postoperative hypoglycemia. This approach may be useful for perioperative glycemic control during third molar extraction.

## Introduction

Intravenous sedation (IVS) has been widely applied for relieving patient anxiety and fear concerning dental treatment, stress accompanying surgery, and similar issues. When administering IVS in a patient, in order to prevent aspiration [1, 2] of the contents of the stomach, patients in our department are instructed to refrain from eating and drinking for 4 h prior to the operation [3–6].

In addition, when performing dental treatment under IVS, several reports [1, 2] have stated that the risk of vomiting and aspiration is the same as that in general anesthesia, and accordingly, restrictions on eating and drinking are necessary [3]. However, these dietary restrictions and the surgery itself cause large fluctuations in the blood glucose level, and Sawano et al. [7] reported that the blood glucose level drops sharply after completion of surgery. Postoperative hypoglycemia is also a factor involved in hemodynamic changes and postoperative complications [8].

The present study investigated the usefulness of glucose administration as a method of maintaining perioperative glycemic control in patients with dietary restrictions during 4 h prior to impacted mandibular third molar extraction under IVS.

## Methods

### Subjects

This study included 54 healthy individuals (18 men, 36 women; mean age, 32.4 years; mean body weight, 55.9 kg; mean body mass index [BMI], 21.1) who were scheduled to undergo impacted mandibular third molar extraction under IVS and who had a preoperative blood glucose level of 70–110 mg/dL. In addition, group assignment was performed using adaptive randomization. The subjects were sufficiently briefed and understood the purpose and details of this study. This study was approved by the Ethical Review Committee of the Nippon Dental University School of Life Dentistry at Niigata (approval number: ECNG-R-327, approval date: December 28, 2017, UMIN Study ID: UMIN000028311). In addition, written informed consent was given.

Based on X-ray examination findings, the embedded status of the wisdom teeth was classified as Class II, Position B, according to the G. B. Winter system of classification. This classification is characterized by a smaller crown length of the mandibular third molar compared with the distal marginal ridge of the mandibular second molar and the ramus of the mandible, with the highest point of the mandibular third molar located below the occlusal surface and above the cervical region of the mandibular second molar [9].

### Measurement environment and conditions

This study was conducted in a quiet room at room temperature (approximately 25 °C, 50% humidity), and the subjects’ compliance with the restrictions regarding eating and drinking during the 4-h period prior to receiving IVS was confirmed.

### Outcomes measured

#### Blood glucose level

**Fig. 1:**
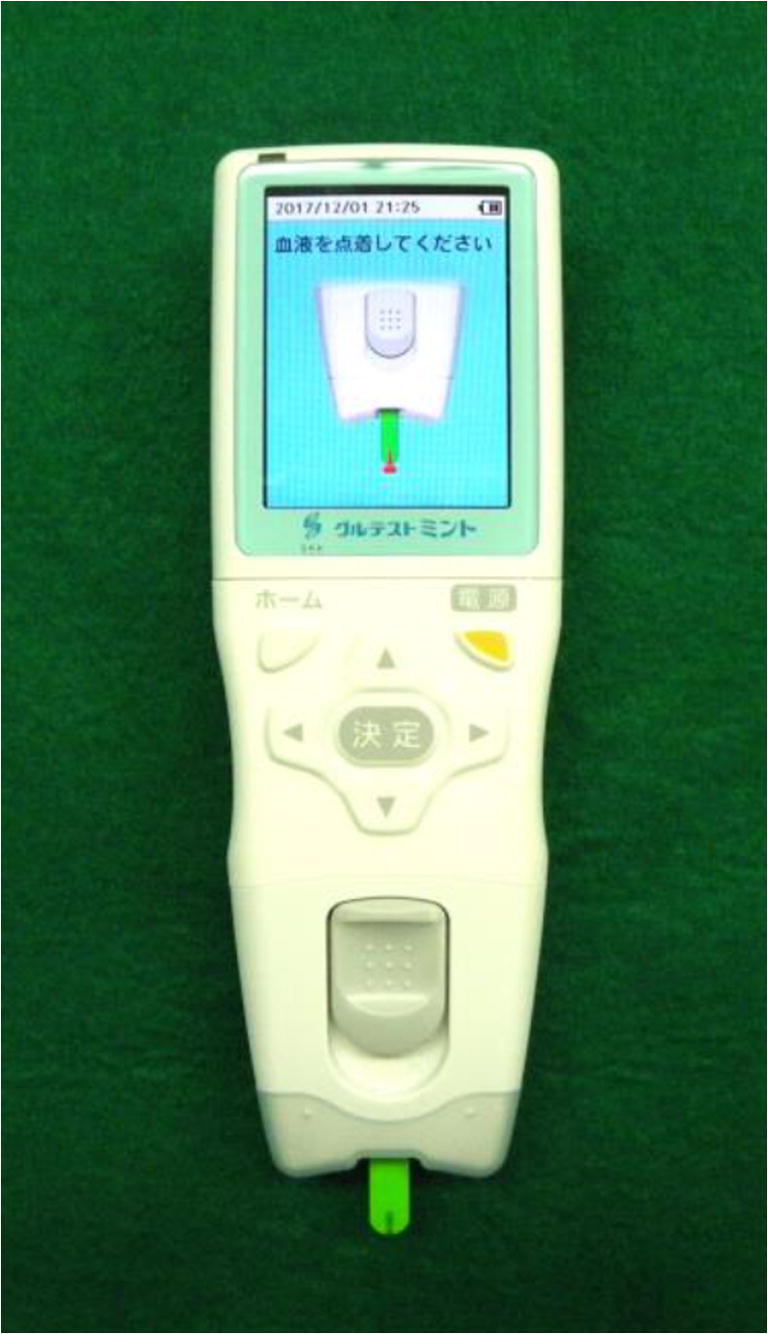
Glutest mint®

The Glutest mint (manufactured by Sanwa Kagaku Kenkyusho Co., Ltd.), a blood glucose-measuring device supporting point-of-care testing (POCT), was used for measurement of blood glucose levels (Fig. 1).

#### Perioperative hemodynamics

For evaluating perioperative hemodynamics, systolic blood pressure, diastolic blood pressure, and heart rate were measured using a BP-A308 biological information monitor (manufactured by Colin Medical Technology Co., Ltd.).

The subjects were placed in a supine position, and access to the forearm vein was secured using a 22G indwelling needle. After the patients rested for 5 min, the blood glucose level was measured using the blood collected from a lateral catheter, and this value was considered to indicate the baseline. Next, after the intravenous administration of 1 g of ampicillin sodium, IVS was started.

Perioperative respiratory parameters/hemodynamics were evaluated on the basis of blood pressure measurements, electrocardiogram results, heart rate, and percutaneous oxygen saturation using the BP-A308 biological information monitor.

Through the lateral catheter, 0.05 mg/kg of midazolam was administered, and the sedation state during surgery was maintained between 3 and 4 on the Ramsay sedation scale [10]. The dosage of midazolam was based on the study by Matsuki et al. [11]. After sedation was achieved, 2.0 mL of 2% lidocaine hydrochloride solution was infused as conduction anesthesia, and 1/80,000 adrenaline added to 3.6 mL of 2% lidocaine hydrochloride was administered as infiltration anesthesia; the operation was initiated 5 min later.

The subjects were divided into three groups: group receiving glucose-free sodium lactate Ringer’s solution (control group, 18 subjects), group receiving 100 mL of 5% glucose solution administered immediately after the local anesthesia (perioperative GL group, 18 subjects), and group receiving 100 mL of 5% glucose solution immediately after the end of surgery (postoperative GL group, 18 subjects). A drip infusion rate of 4 mg/kg/min was used in all groups.

**Fig. 2:**
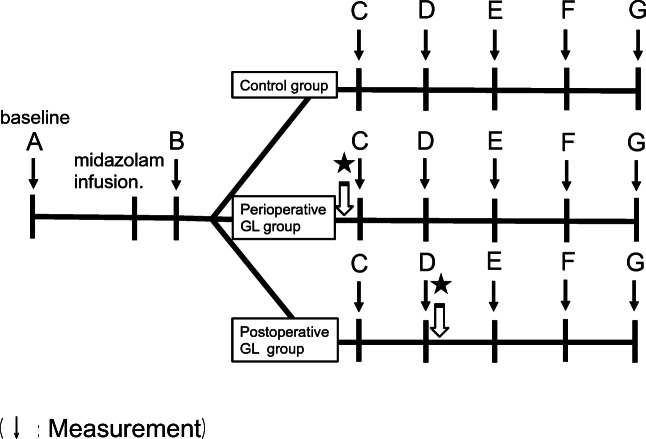
Timetable of the tests. A, initiation of the measurements; B, post local anesthesia; C, start of extraction; D, end of extraction; E, 30 min after completion of the surgery; F, 60 min after completion of the surgery; G, 90 min after completion of the surgery; star, infusion of 5% glucose

Cases where the patient received additional administration of 1/80,000 adrenaline added to 2% lidocaine hydrochloride or where the operative time was longer than 40 min were excluded from the analysis for this study, and all procedures were performed by oral surgeons with over 5 years of clinical experience to ensure that all treatments were performed as consistently as possible. At the end of tooth extraction, 50 mg of flurbiprofen axetil diluted in 50 mL of physiological saline was intravenously administered for postoperative analgesia. Each parameter was measured seven times: before surgery (baseline); after administration of local anesthesia (LA); at the start of surgery (ope start); at the end of surgery (ope end); and 30 min, 60 min, and 90 min after completion of the surgery (Fig. 2).

The SPSS Statistics version 22 statistical software (SPSS, IBM Japan, Japan) was used for statistical analysis. For the change in the blood glucose level with respect to the baseline, a repeated two-way analysis of variance (ANOVA) was performed, and Bonferroni’s multiple comparisons test was performed when the interaction was significant. In addition, multiple comparisons between groups were made at the same time point, and the level of statistical significance was set to *p* < 0.01 or *p* < 0.05. The age, body weight, BMI, and operation time of each group were analyzed by one-way ANOVA, and the level of statistical significance was set to *p* < 0.05.

## Results

There was no significant difference among the groups with regard to age, body weight, BMI, and preoperative blood glucose level (Table 1). In addition, the mean operative time was 27.9 min ± 5.6 min, and no significant differences were observed between groups. In all subjects, there was no abnormality in perioperative electrocardiogram, and saturation of percutaneous oxygen was 95% or more.

**Table 1:** Age, body weight, BMI, and preoperative blood glucose levels

	Perioperative		Postoperative	
	Control group	GL group	GL group	
Age (year)	30.1±9.2	32.6±6.4	34.7±7.1	N.S.
Weight (kg)	54.4±11.5	51.0±8.2	56.2±8.9	N.S.
BMI (kg/m^2^)	21.5±3.7	19.7±2.4	21.0±1.7	N.S.
Male/Female	4/14	5/13	9/9	N.S.
Blood glucose level (baseline, mg/dL)	91.5±9.34	91.5±9.12	89.6±15.5	N.S.
Operative time (min)	27.2±5.2	27.8±6.0	28.9±5.6	N.S.

Mean ± SD

*N.S.* not significant

### Blood glucose level

#### Intragroup comparison

##### Control group (Fig. 3, i)

The baseline value was 91.5 ± 9.34 mg/dL, and there was no significant difference from the level at administration of local anesthesia to 60 min after the operation, but a significant decline was observed 90 min after the operation; the blood glucose level at that time was 85.0 ± 7.54 mg/dL (*p* < 0.05).

**Fig. 3:**
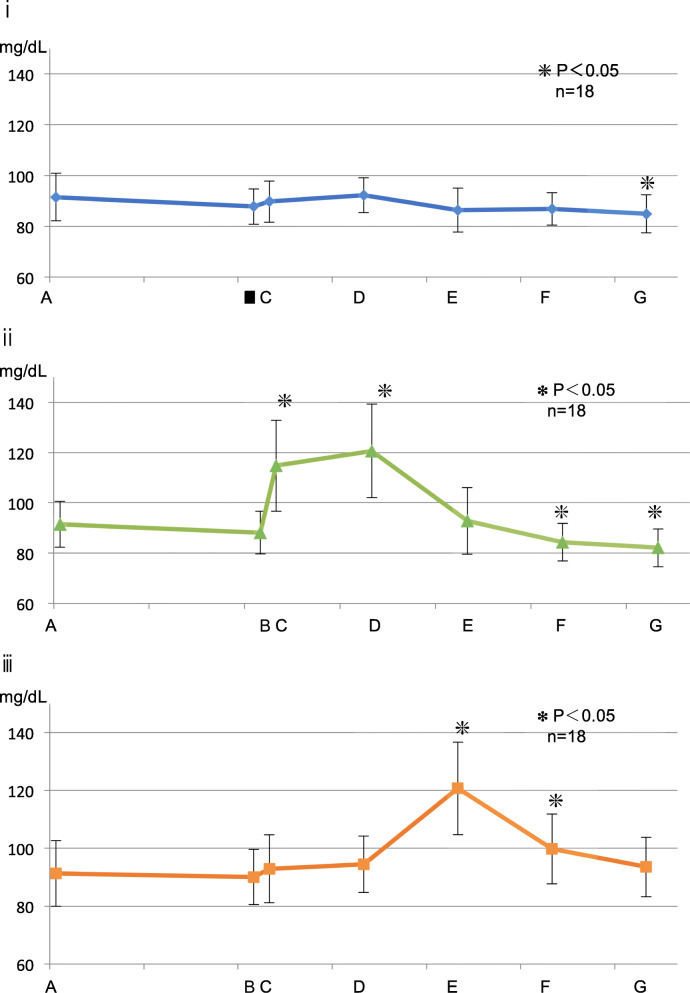
Fluctuation in the blood glucose level. (i) Control group. (ii) Perioperative group. (iii) Postoperative group. The asterisks indicate a significant difference from the baseline (A). Data represent the mean ± SD

##### Perioperative GL group (Fig. 3, ii)

The baseline value was 91.5 ± 9.12 mg/dL, which became 114.7 ± 18.16 mg/dL after the start of surgery and further increased to 120.7 ± 18.61 mg/dL after the operation was completed; however, the blood glucose level subsequently decreased to 84.3 ± 7.43 mg/dL, and further fell to 82.2 ± 7.56 mg/dL 90 min after completion of the surgery (*p* < 0.05).

##### Postoperative GL group (Fig. 3, iii)

The baseline value was 89.6 ± 15.5 mg/dL, which showed no significant fluctuation until the end of surgery; however, it increased to 120.9 ± 16.71 mg/dL 30 min after the operation and 99.0 ± 13.66 mg/dL after 60 min (*p* < 0.05). No significant difference was observed at 93.0 ± 13.02 mg/dL 90 min after the operation.

#### Intergroup comparison (Fig. 4)

No significant difference was observed in the baseline value; however, a significant difference was observed between the control group and the postoperative GL group 90 min after the operation, and a significant difference was observed between the perioperative GL group and the postoperative GL group (*p* < 0.05 for both). No significant difference was observed between the control group and the perioperative GL group 90 min after the operation.

**Fig. 4:**
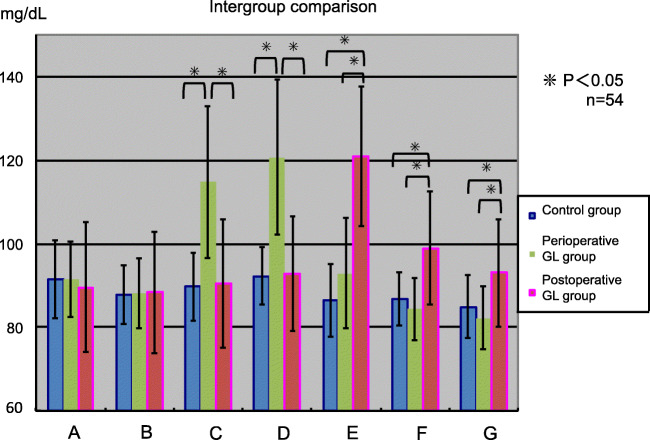
Fluctuation in blood glucose level (intergroup comparison). The asterisks indicate a significant difference between the groups

### Systolic blood pressure and diastolic blood pressure

#### Trends for all 54 subjects (Fig. 5)

Compared with the preoperative systolic blood pressure of 116.1 ± 13.79 mmHg, the following changes were noted: 103.7 ± 11.27 mmHg 30 min after completion of the operation, 104.3 ± 10.88 mmHg 60 min after completion, and 107.5 ± 11.66 mmHg 90 min after completion (*p* < 0.05).

**Fig. 5:**
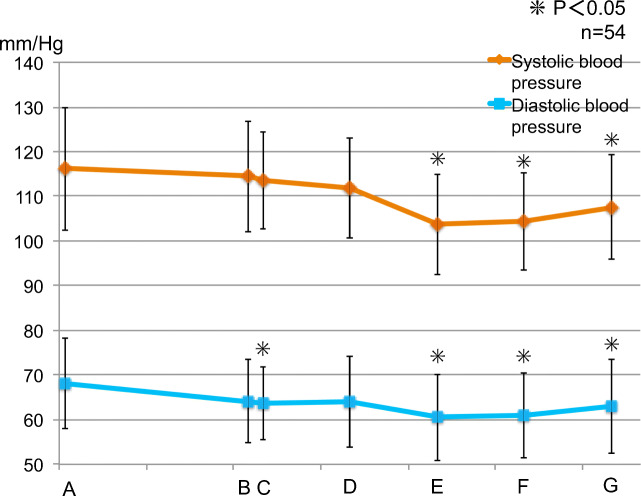
Fluctuation in systolic blood pressure and diastolic blood pressure of all subjects. The asterisks indicate a significant difference from the baseline (A). Data represent the mean ± SD

Compared with the preoperative diastolic blood pressure of 68.1 ± 10.09 mmHg, it was 63.7 ± 8.21 mmHg after the start of surgery, 60.5 ± 9.66 mmHg 30 min after completion, 61.0 ± 9.54 mmHg 60 min after completion, and 63.0 ± 10.56 mmHg 90 min after completion (*p* < 0.05).

#### Intergroup comparison (Fig. 6)

No significant difference was observed at all measurement points between the groups.

**Fig. 6:**
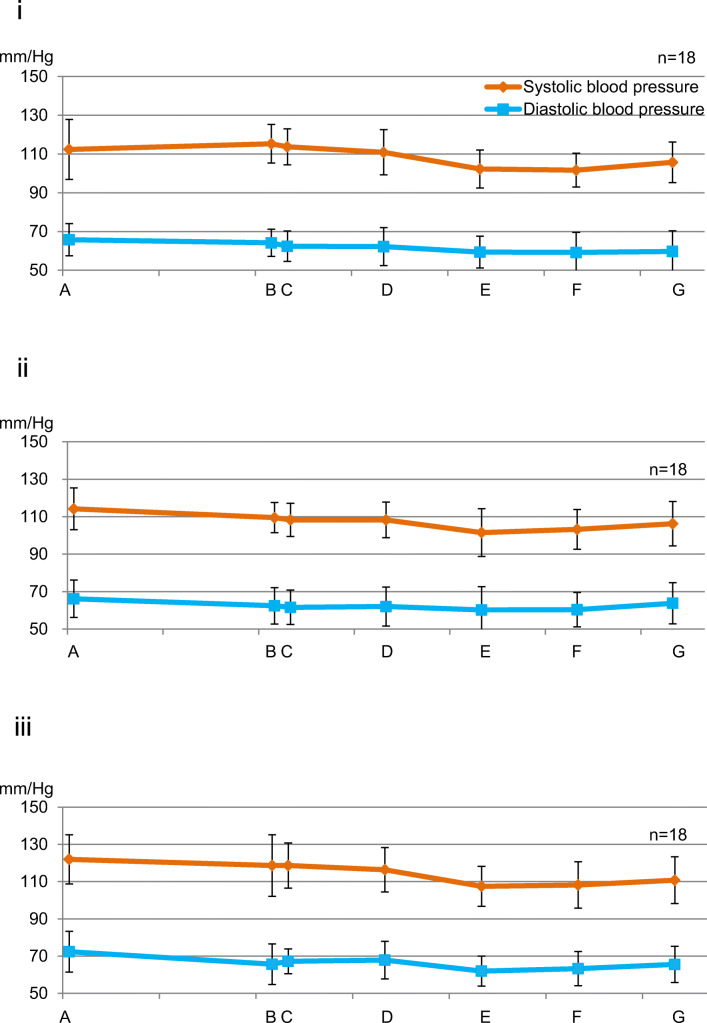
Fluctuation in systolic blood pressure and diastolic blood pressure (between groups). (i) Control group. (ii) Perioperative group. (iii) Postoperative group. There was no significant difference at all measurement points. Data represent the mean ± SD

### Heart rate

#### Trends for all 54 subjects (Fig. 7)

Heart rate increased to 87.1 ± 12.74 beats/min after local anesthesia compared to 72.8 ± 10.79 beats/min before the surgery; further, the rate was 82.0 ± 10.05 beats/min after the start of the surgery, 78.3 ± 10.81 beats/min after the completion of the surgery, 66.7 ± 9.34 beats/min 60 min after the completion of the surgery, and 66.9 ± 9.91 beats/min 90 min after the completion of the surgery (*p* < 0.05).

**Fig. 7:**
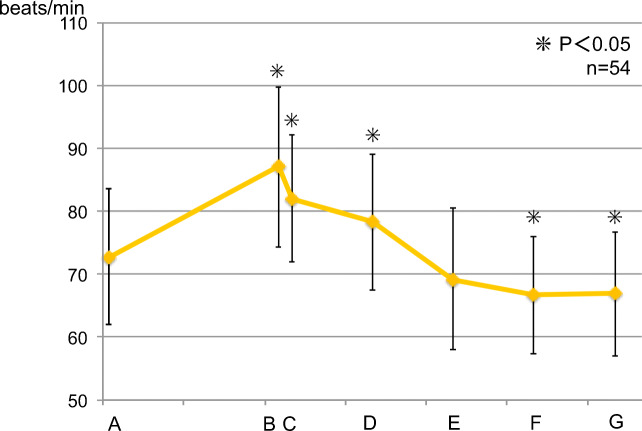
Fluctuation in heart rate of all subjects. The asterisks indicate a significant difference from the baseline (A). Data represent the mean ± SD

#### Intergroup comparison (Fig. 8)

No significant difference was observed at all measurement points between the groups.

**Fig. 8:**
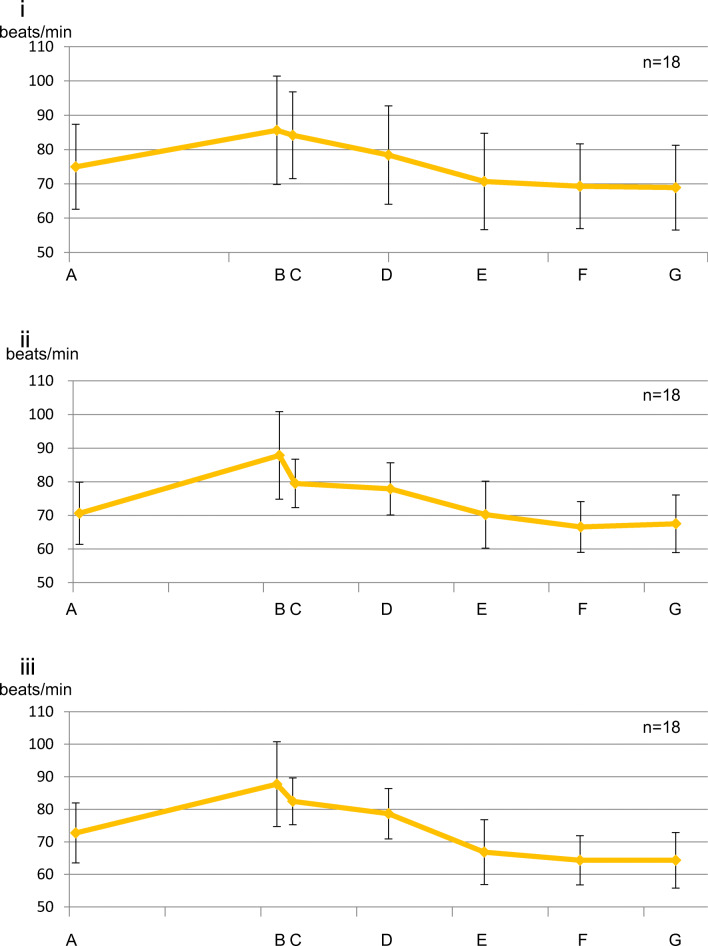
Fluctuation in heart rate (between groups). (i) Control group. (ii) Perioperative group. (iii) Postoperative group. There was no significant difference at all measurement points. Data represent the mean ± SD

## Discussion

In this study, we investigated the usefulness of glucose administration for maintaining perioperative glycemic control in patients with dietary restrictions during 4 h prior to impacted mandibular third molar extraction under IVS. The blood sugar level decreased within the standard range in the control group and the perioperative GL group 90 min after the operation, compared with the preoperative blood glucose level; however, in the postoperative GL group, blood glucose levels were similar to the preoperative levels.

The subjects’ baseline blood glucose levels were 70–110 mg/dL; this range was selected based on the diagnosis of hypoglycemia when the fasting blood glucose level is less than 70 mg/dL, and borderline-type diabetes when the fasting blood glucose level is higher than 110 mg/dL according to the Japanese Clinical Practice Guidelines for Diabetes 2016 published by the Japan Diabetes Society.

In this study, we used a 5% glucose solution with a low risk of hyperglycemia and expected a gradual increase in blood glucose level. The administration rate was set at 4 mg/kg/min with reference to the report of Rosmarin et al. [12]. This administration rate has no side effects such as hyperglycemia, and the glucose is fully utilized.

During tooth extraction and dental treatment, the blood glucose level increases due to the action of adrenaline present in the local anesthetic solution [7, 13, 14], but various psychological stressors such as physical stress, anxiety regarding treatment, and feelings of insecurity have also been reported to cause an increase in blood glucose levels during surgery [7, 14, 15]. In addition, it is known that blood levels of compounds such as catecholamines, cortisol, glucagon, and growth hormone become elevated due to stress reactions, potentially leading to the onset of hyperglycemia [14, 16–18]. However, other reports have stated that this can be suppressed through perioperative pain control measures [18, 19].

In this study, the postoperative blood glucose level in the control group decreased significantly within the standard range. It was thought that increases in the levels of substances such as catecholamines and cortisol due to sympathetic nerve stimulation were suppressed as patients’ psychological stress was alleviated through the combined use of IVS and pain control by local anesthesia. The subjects’ blood glucose levels did not rise despite the use of an adrenaline-containing local anesthetic solution, which may be attributed to the combination of alleviation of psychological stressors and dietary restriction having a greater effect on blood glucose level compared with the blood glucose-increasing effect of adrenaline. As the subjects of this study were adults without any systemic disease, the decrease in blood glucose level was within the normal range, and no critical deterioration was observed. However, the postoperative blood glucose levels showed a clear reduction even with restriction on eating and drinking for the relatively short period of 4 h.

Blood glucose levels in the perioperative GL group decreased significantly between 60 and 90 min after surgery completion. In healthy adults without diabetes and other diseases, the pancreatic islets’ β cells instantly secrete insulin as the blood glucose level rises [20, 21]. This study also found that as a result of additional secretion of insulin from the start of glucose administration to 90 min after the operation, and because there were no invasive procedures or stressors and the sedation/analgesia was maintained, catecholamine and cortisol hormones that elevate blood glucose levels were suppressed, resulting in a significant decrease in blood glucose level.

In the postoperative GL group, blood glucose levels increased transiently after glucose administration and subsequently declined, but 90 min after operation, the level recovered to the baseline value and remained stable. This is believed to be due to the additional secretion of insulin after glucose administration as described above, which resulted in regulation of blood glucose in the body to the baseline value in the normal range. In the intergroup comparison, the blood glucose level in the postoperative GL group was significantly higher than that of the control group and perioperative GL group 90 min after the operation.

Since systolic blood pressure, diastolic blood pressure, and heart rate decreased after the end of surgery compared to their respective preoperative levels, sedation was maintained without the need for invasive procedures, and the patients were believed to be in a stress-free state. According to a previous report, the patient’s hemodynamic state is more closely related to the stress caused by surgery than to the amount of local anesthesia to which adrenaline is added during wisdom tooth extraction [22]. In the present study, sedation was continuing, so there was no increase in systolic and diastolic blood pressure after administration of local anesthesia. However, the heart rate increased after the administration of local anesthesia until the end of surgery. This indicates that adrenaline β1 action increases the heart rate even in a stress-free situation in a sedative state.

This study revealed that the blood glucose level after induction of IVS decreases within the standard range following dietary restriction during the 4 h prior to the start of surgery. Further, administration of glucose after completion of surgery was useful for preventing postoperative hypoglycemia. Nevertheless, if pain and swelling persist after surgery and subjects have difficulty ingesting, there is a risk of further lowering of blood glucose levels. As such, in future studies, it will be necessary to determine the optimal concentration of glucose to be administered and the rate of administration necessary to prevent hypoglycemia. In addition, patients with a history of diabetes often exhibit worsening glycemic control, and clinical manifestations such as hypoglycemia coma are a concern [8], and the results of this study may represent basic research for effective perioperative management of diabetic patients in whom dental procedures, such as extraction of impacted third molars, are indicated.

However, since only the timing of glucose administration is studied in this research, it is necessary to repeat the research including the concentration of glucose and so on.

## Conclusions

In patients who had restricted eating and drinking 4 h prior to the extraction of the impacted mandibular third molar under IVS, the blood glucose level dropped during the perioperative period but remained within the standard range.

It was also revealed that in healthy adults, administration of 100 mL of 5% glucose solution immediately after the end of surgery can prevent the lowering of the blood glucose level thereafter.
